# Looking beyond LCI: Multiple breath washout phase III slope derived indices and their application in chronic respiratory disease in children

**DOI:** 10.1002/ppul.27177

**Published:** 2024-07-19

**Authors:** Mollie Riley, Michele Arigliani, Gwyneth Davies, Paul Aurora

**Affiliations:** ^1^ Infection, Immunity and Inflammation Research and Teaching Department UCL Great Ormond Street Institute of Child Health (UCL GOS ICH) London UK; ^2^ Heart and Lung Directorate Great Ormond Street Hospital for Children NHS Foundation Trust London UK; ^3^ Department of Respiratory Paediatrics Royal Brompton Hospital London UK; ^4^ Population, Policy and Practice Research and Teaching Department UCL GOS ICH London UK

**Keywords:** children, cystic fibrosis, multiple breath washout, phase III slope analysis, ventilation inhomogeneity

## Abstract

The multiple breath washout (MBW) test is widely reported in the context of Lung Clearance Index (LCI). LCI reflects global ventilation inhomogeneity but does not provide information regarding the localization of disease along the respiratory tree. The MBW‐derived normalized phase III slope (S_nIII_) indices (S_cond_ and S_acin_), instead, can distinguish between convective‐dependent and diffusion‐convection‐dependent ventilation inhomogeneity considered to occur within the conductive and acinar airways, respectively. In cystic fibrosis, S_cond_ tends to become abnormal even earlier than LCI and spirometry. The value of S_cond_ and S_acin_ in clinical practice has been recently explored in other respiratory conditions, including asthma, primary ciliary dyskinesia, bronchopulmonary dysplasia, bronchiolitis obliterans, and sickle cell disease. In this narrative review we offer an overview on the theoretical background, potentialities, and limitations of S_nIII_ analysis in children, including challenges and feasibility aspects. Moreover, we summarize current evidence on the use of S_nIII_‐derived indices across different groups of pediatric chronic respiratory disease and we highlight the gaps in knowledge that need to be addressed in future studies.

## 1 INTRODUCTION

Multiple breath washout (MBW) is a type of inert gas washout test that measures ventilation distribution, the efficiency of gas mixing, dead space, and resting lung volume. Inert marker gases used include sulfur hexafluoride (SF_6_), helium, or resident nitrogen (N_2_) displaced by breathing 100% oxygen (O_2_). Simultaneous washout of two marker gases with differing molecular diffusivities (SF_6_ and helium) may offer more specific information on peripheral ventilation distribution.^1^

Most of the research and clinical application of MBW is in pediatric cystic fibrosis (CF); however, its use is being increasingly extended to other respiratory conditions like primary ciliary dyskinesia (PCD) and asthma. The test is particularly attractive in pediatrics due to its superior sensitivity at identifying early CF lung disease and greater feasibility across a wider age range compared to spirometry.^2^, ^3^ The lung clearance index (LCI) is the most commonly reported MBW outcome, representing the degree of ventilation inhomogeneity (VI) in the lungs. An abnormal LCI can result from diverse pathologies, including patchy airway disease, patchy changes in lung compliance, or disruptions of peripheral lung architecture.

However, the LCI determines the global VI but does not provide additional information on localization of disease or gas transport processes that generate VI.

**Figure 1:**
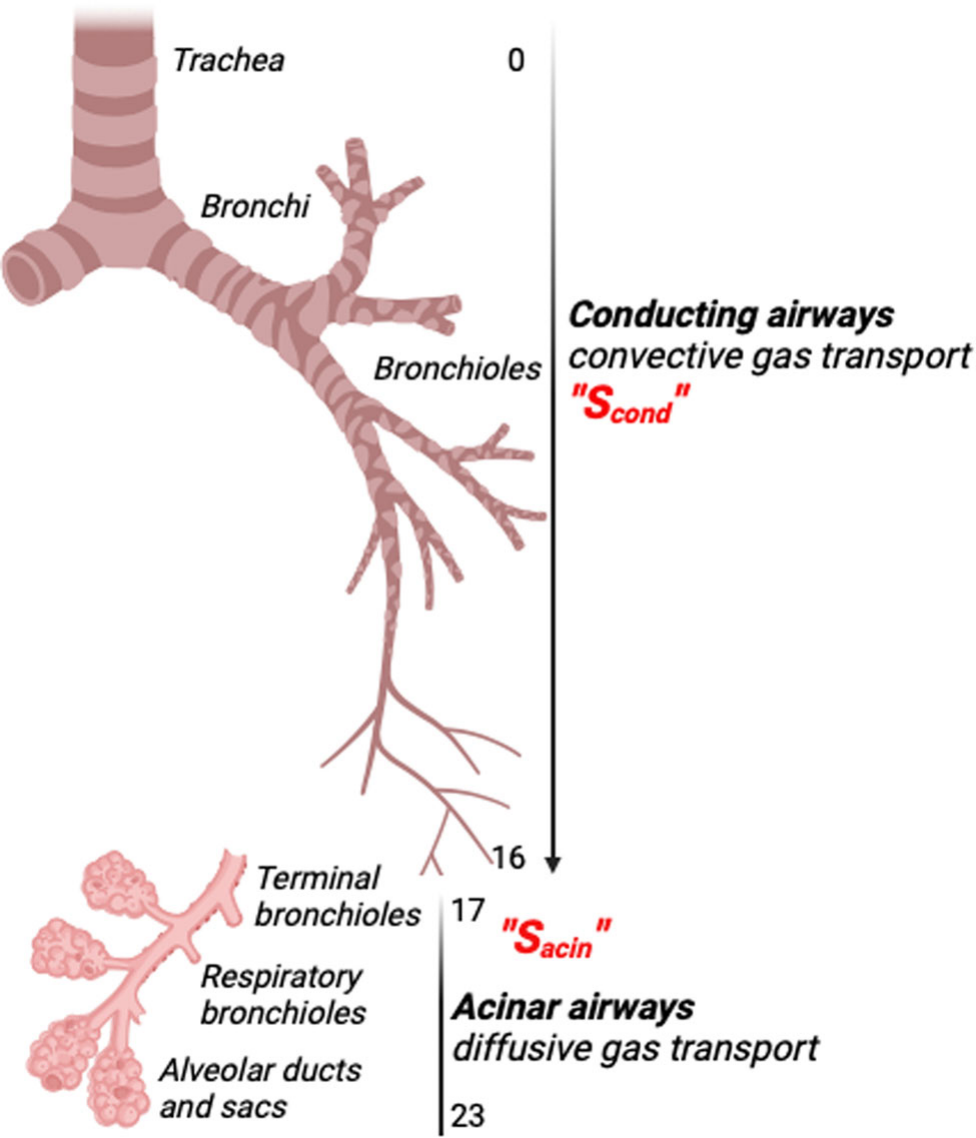
A schematic model of the branching airway tree with the conducting and acinar airways labeled (created with BioRender.com).

The analysis of the progression of the normalized phase III slope (S_nIII_) of every breath through the course of the MBW can identify and separate the physiological mechanisms of VI, which in turn may reflect structural changes within the lung. This is achieved by calculating two separate indices. S_cond_ is predicted to reflect convection‐dependent ventilation inhomogeneity (CDI) arising within the conductive airways (Figure 1), while S_acin_ is predicted to reflect diffusion–convection interaction‐dependent ventilation inhomogeneity (DCDI), arising in the healthy lung, at the entry of the acinar region (generations 17–23, Figure 1).^4^, ^5^

S_nIII_ indices have the potential to identify and track early abnormalities in the conductive airways or in the lung periphery, and as such could have multiple clinical applications. Indeed, assessment of S_cond_/S_acin_ may facilitate clustering into distinct phenotypes, potentially paving the way for guiding and monitoring personalized treatment therapies in the future. This approach has already been demonstrated in adult asthma.^6^ However, the use of S_cond_ and S_acin_ to date has been mostly limited to research and there is the need for wider validation in clinical settings, especially in children.

In this narrative review, we outline the use of S_nIII_ analysis (S_cond_ and S_acin_) in pediatrics including theoretical considerations and limitations. We also provide an overview on the existing literature that incorporates S_cond_ and S_acin_ in the assessment of conductive and acinar airway impairment in chronic respiratory diseases in children. This review examines the clinimetric properties of S_cond_ and S_acin_ concerning pediatric patients and highlights the existing areas where further research is needed.

A literature search was conducted in Ovid MEDLINE using search terms related to S_nIII_ analysis, including “S_cond_,” “phase III slope,” “regional ventilation inhomogeneity,” “S_nIII_,” and “conductive ventilation inhomogeneity.” These terms were combined with terms such as “child*,” “preschool,” and “paediatric*.mp.” Papers published from 2007 until September 2023 were included.

## 2 THE PHYSIOLOGICAL BASIS OF PHASE III SLOPE ANALYSIS

**Figure 2:**
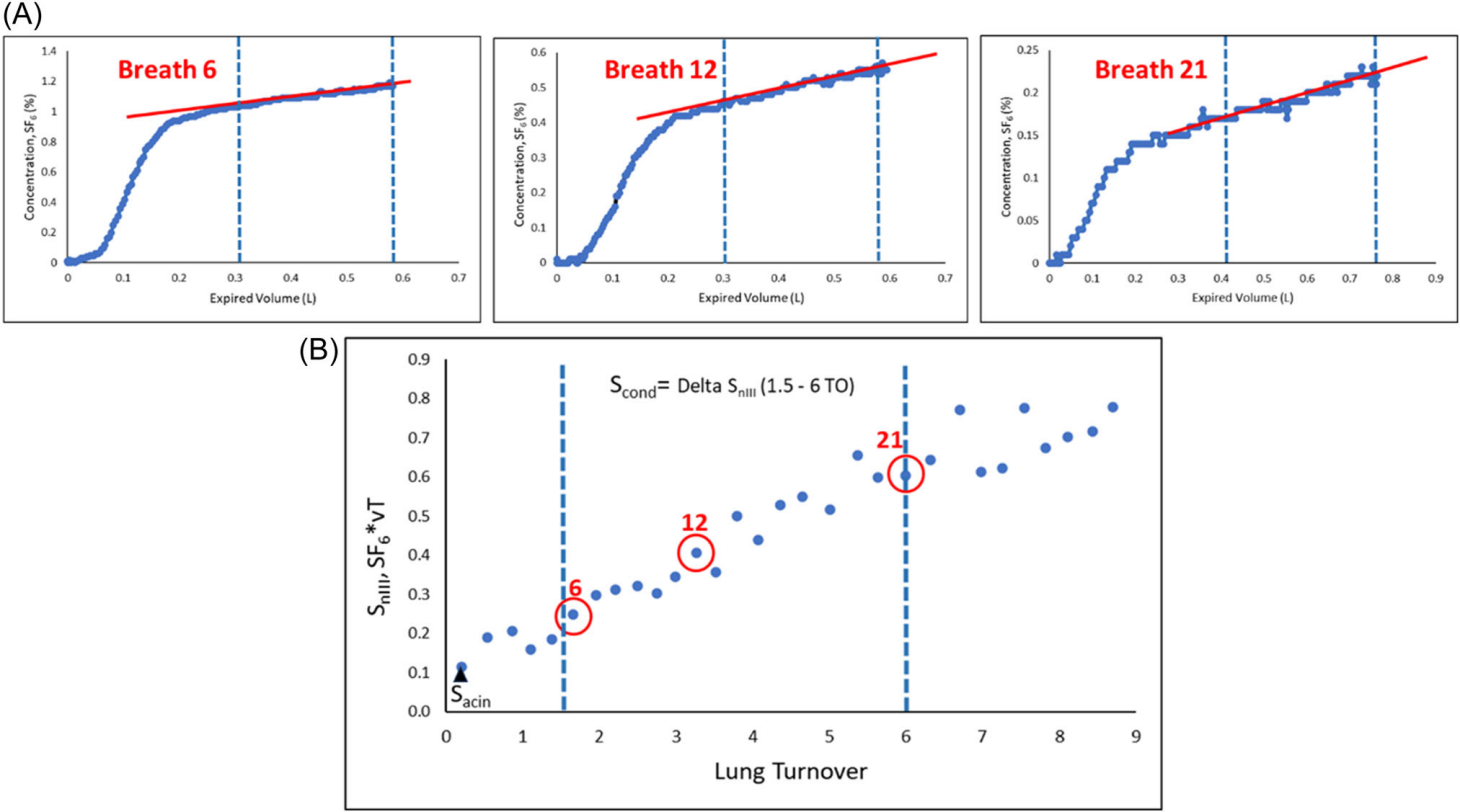
Graphic representation of S_cond_ from one multiple breath washout (MBW) trial. Three breaths at different stages of an SF_6_ MBW are displayed. Figure 2A displays the expirogram of each breath (SF_6_ concentration against volume [L]). As the SF_6_ concentration decreases and expiratory volume changes over the course of the washout, the scaling of the *x* and *y*‐axes are not uniform. The phase III slope is numerically the coefficient of the linear regression (red line) of the tracer gas concentration (*y*‐axis) versus expired volume (*x*‐axis) in the alveolar phase III (50%–95% of the expired volume, delimited by vertical blue lines). The alveolar slope is divided by mean expired SF_6_ concentration over the phase III interval and multiplied by the expiratory tidal volume of the breath in liters, to give a final number which represents the normalized alveolar slope (S_nIII_).

As an introductory overview, a graphic explanation of S_nIII_ indices is given in Figure 2. The lungs have evolved into a branching network of airways that extend out to a huge periphery, facilitating effective gas mixing and exchange. Gas is transported within the lung mainly by convection (i.e., driven by differences in pressure gradients) in the conductive airways (generations 0–16) and by diffusion (i.e., driven by differences in gas concentration) in the intra‐acinar zone (generations 17–23).

Differences in the specific ventilation between lung units sharing a branch point in the conducting airways and flow asynchrony between these units during exhalation may occur due to heterogeneous reduction of the lumen (e.g., mucus plugging) or differences in the compliance of these units subtended to the branching point.

In these circumstances, the inert gas from poorer ventilated lung units reaches the mouth later in the expiration than the gas from better ventilated units and thus contributes later to the alveolar phase III of the expirogram. As the tracer gas is cleared, the discrepancy in tracer gas concentration between well‐ventilated and poorly ventilated units becomes greater resulting in an increasing steepness of S_nIII_ over the consecutive breaths (Figure 2A). This is the CDI measured by S_cond_ ^5^ and is predicted to increase linearly throughout the course of the MBW.^4^, ^7^ In the presence of lung disease leading to differences in specific ventilation, this pattern can be easily seen through the course of the MBW. In healthy lungs, however, the phase III slope of each breath is almost flat (although is still positive) and changes very little throughout a washout.

Further into the lung periphery, the contribution of convection to gas transport decreases, and the contribution of molecular diffusion greater. The region where both mechanisms provide similar contributions is termed the “diffusion‐convection front.” Generally, molecular diffusion will tend to counter the inhomogeneity created by convection. However, additionally, if there are differences in the cross‐sectional areas or subtended lung volumes of the intra‐acinar airways sharing branching points at this level, this will result in an increased phase III slope in the first expiratory breath. Diffusion–convection interaction will then contribute progressively less to the positive slope of subsequent breaths, and eventually reach asymptote, as differences in gas concentration between the intra‐acinar lung units are eliminated by molecular diffusion.^4^, ^7^ This is the DCDI measured by S_acin_.^5^ In adult humans this asymptote is predicted to occur by the fifth breath of the washout. This point can also be expressed as 1.5 lung volume turnovers (TO), where TO is calculated as the cumulative expired volume (CEV)/functional residual capacity (FRC). It is important to note that the diffusion‐convection front is a physiological rather than anatomical location. In the healthy adult human lung, this front is predicted to be located at the acinus entrance, hence the use of the index S_acin_ to quantify DCDI.

In Figure 2B the S_nIII_ values of the washout breaths are plotted against their corresponding lung volume turnover (TO; 1 TO = CEV that equals the FRC). The specific breaths in Figure 2A are circled in red in Figure 2B. S_cond_ reflects convection‐dependent inhomogeneity (CDI) arising within the conductive airways proximal to acinar zones. It is obtained by the calculated S_nIII_ increase between 1.5 and 6.0 TO of the washout. The choice of 1.5 TO is to ensure no further contribution of DCDI is present and the upper limit of 6 TOs has been shown to be most appropriate recently.^8^ S_acin_, instead, is intended to reflect DCDI at the entry of the acinus. Approximately 80% of the slope of the first breath is generated by DCDI. S_acin_ is calculated by computing the S_nIII_ of the first breath of the washout minus the S_cond_ contribution to its S_nIII_ value.^5^

## 3 CHALLENGES WITH CLINICAL IMPLEMENTATION OF S_NIII_ ANALYSIS

Although a wider application of S_nIII_ analysis may appeal, there are several challenges. Some apply to all subjects; others are specific to children. These can be considered as issues around standardization and quality control (QC), invalidity of these indices in the presence of severe inhomogeneity, equipment issues, lack of reference data, and interpretation of S_nIII_ indices in children.

### 3.1 Acceptability criteria for S_nIII_ analysis

When S_cond_/S_acin_ were originally proposed by Verbanck et al.,^5^ a fixed 1‐L breathing protocol was used to perform MBW to allow for clear identification of the alveolar plateau of each breath, from which the S_nIII_ is derived. However, this method is not possible in children as it falsely elevates LCI and S_cond_.^9^ Instead, the test is carried out during spontaneous tidal breathing (usually with distraction, e.g., cartoon videos).^10^

**Figure 3:**
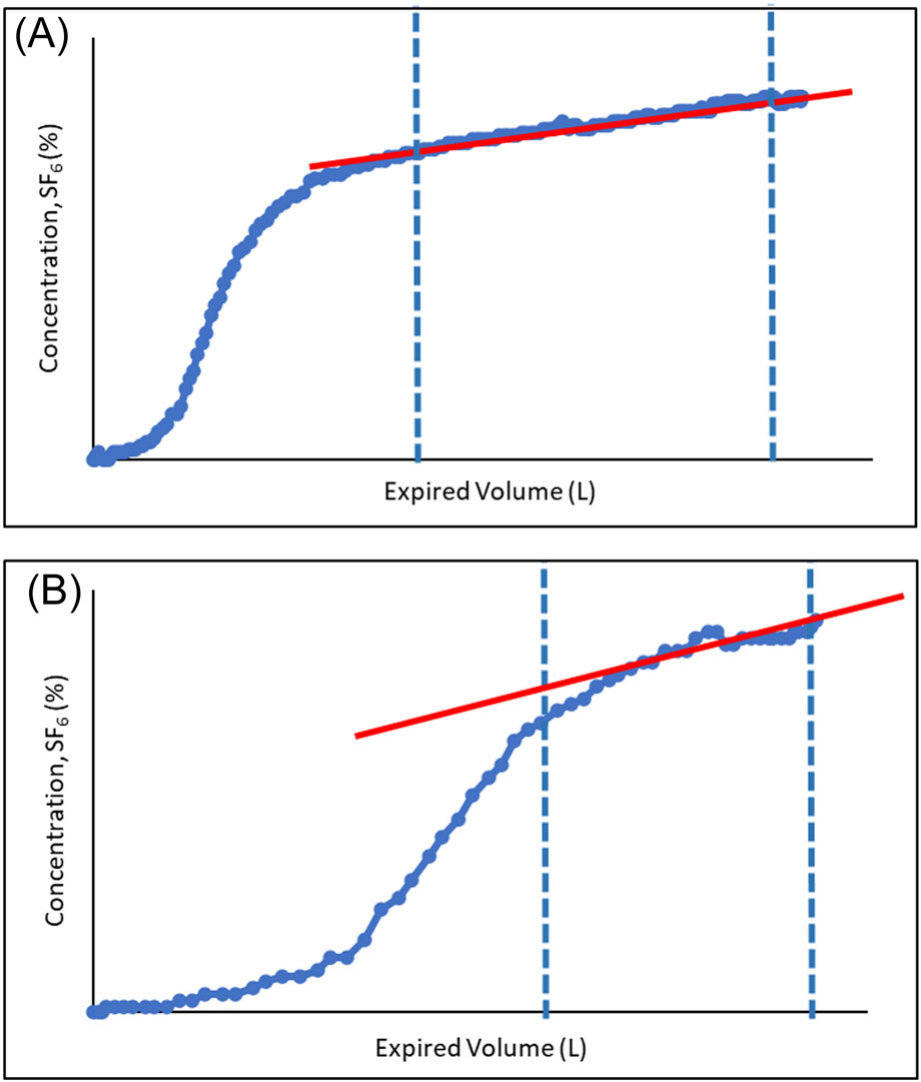
Two expirograms (SF_6_ concentration against volume [L]) representing two individual breaths of a washout from adolescents with CF. Breath (A) has an adequate expired vT with a sufficient phase III portion of the breath regressed. Breath (B) has a small expired vT with no clear phase III portion of the breath and is not suitable for S_nIII_ analysis.

To account for the higher breath‐by‐breath variability in tidal volume (vT), in children the S_nIII_ is multiplied by the expiratory tidal volume of the breath (vT × S_nIII_). However, pediatric washouts often have several breaths with relatively small vT (generally <10 mL/kg) and no clear phase III portion (phase III volume <50% of the entire vT) or, conversely, with large vT breaths (generally >15 mL/kg) where the phase III volume represents >75% of the vT, which also may not be suitable for S_nIII_ analysis (Figure 3).

Breaths with irregular expiration (e.g., swallows) or noise (e.g., electronic or oscillations) should also be excluded from S_nIII_ analysis.

Based on the current ERS/ATS consensus statement recommendations, S_cond_ and S_acin_ should then be calculated, respectively, only for trials with a first breath of adequate quality and with at least two‐ thirds of S_nIII_ values left after analysis.^10^ S_nIII_ indices should be preferably reported for MBW tests with three trials acceptable for S_nIII_ analysis. However, considering the technical challenges and the higher failure rate in children, S_acin_ and S_cond_ have been also reported as the average of at least two acceptable trials rather than 3.^11^, ^12^

### 3.2 Variability

S_cond_/S_acin_ have large intra‐test^13^, ^14^, ^15^ and inter‐test variability^16^ compared to LCI, likely due to their susceptibility to changes in vT. Moreover, variability appears to be more pronounced in health than in disease.^13^, ^14^, ^15^ Supporting Information S1: E‐Table 1 provides more information on S_cond_/S_acin_ variability reported in studies. Investigation into reproducibility within and between children is complicated by the feasibility of testing times that would be required. Current guidelines lack criteria on reproducibility of S_nIII_ outcomes.^10^ Defining what constitutes a “clinically meaningful change” of S_nIII_ outcomes presents significant challenges but it is arguably desirable before these outcomes can be implemented in clinical practice.

### 3.3 Manual versus automated quality control

The traditional approach to S_nIII_ analysis involves a manual breath‐by‐breath QC performed by the operator. The EasyOne Pro, MBW Module (ndd Medical Technologies) and the Exhalyzer D® (Eco Medics AG) are two commercially available MBW devices. The device most used for N_2_MBW, Exhalyzer D® running Spiroware® software, offers an automated, software‐based QC for derivation of S_acin_ and S_cond_, based on the exclusion of S_nIII_ values from breaths with vT deviating >25% from the median vT of the trial. This algorithm aims to exclude irregular breaths to ensure robust fitting quality, similar to the traditional approach, and is quicker and less subjective than manual QC. Moreover, a study comparing manual and automated QC methods for S_nIII_ analysis showed comparable outcomes for S_cond_ in 35 school‐age children with CF.^17^ However, the criterion of eliminating breaths deviating >25% from the median vT applied by the latter does not have a firm physiological basis and does not consider other important parameters that can affect S_nIII_ (especially in younger children), like the phase III volume/vT ratio and the presence of irregular expiration and/or oscillations.

### 3.4 S_nIII_ indices are not valid in the presence of high ventilation inhomogeneity

Modeling studies predict that S_cond_ reaches an asymptote during the course of a washout. In the majority of subjects this will happen very late in the washout—beyond the point where recording is normally terminated—and therefore will not affect calculations. However, this asymptote occurs early in the presence of high VI, with underestimation of S_cond_.^18^ Studies have shown that S_cond_ is not reliable in people with advanced CF lung disease^13^, ^19^ and suggested that the asymptote is reached at around 0.150.^13^, ^14^, ^20^ This is intuitive as S_cond_ measures the progressive increase of S_nIII_ steepness, which occurs because the less well‐ventilated lung units are washed out later over the course of the trial. However, in the presence of high VI, like in advanced CF lung disease, the S_nIII_ will be steep from the first few breaths of the washout, with little room for further progression of the S_nIII_ between 1.5 and 6 TO (when S_cond_ is measured). Verbanck et al.^19^ proposed the alternative indices S_cond*_ and S_acin*_ to capture regional VI in more advanced disease. S_cond_* is measured between the second breath and third lung TO, assuming that also in patients with high VI, there will be some progression of S_nIII_ early during the washout. Observational studies in adults and children have effectively shown that S_cond*_ and S_acin*_ perform better than S_cond_ and S_acin_ in people with advanced CF lung disease as assessed by LCI ^19^, ^20^ and by advanced chest imaging.^21^ The use of S_cond*_ and S_acin*_ is generally recommended in the presence of LCI ≥ 9.^20^

### 3.5 Reference values

While some reference values exist in adults,^22^, ^23^ currently, there are no published reference values for S_cond_ and S_acin_ in children, further limiting their use in clinical practice. This highlights the need of enrolling a control group of healthy subjects matched by age and sex when using S_nIII_ analysis for research purposes. Like LCI, reported S_nIII_ indices may vary between devices, software (and settings), inert marker gases, and methodology used for their calculation. These may limit comparisons between sites. The Global Lung Initiative (GLI) taskforce plan to soon publish normative values for MBW (LCI and FRC); however, these will not include S_cond_ and S_acin_.

### 3.6 Equipment issues

S_nIII_ analysis is often performed “offline” by running recordings on custom‐designed software such as LabView® (National Instruments), WBreath© (ndd Medical Technologies), or TestPoint™ (Capital Equipment Corp.). However, custom‐designed software is not easily accessible and requires specialist training. The Exhalyzer D® software Spiroware® reports results for S_cond_ and S_acin_ in real‐time, immediately on completion of a washout trial. This potentially could lead to inexperienced operators reporting erroneous results before QC. Additionally, Spiroware® reports end‐results for S_cond_/S_acin_ as the mean of three trials, instead of from all data pooled from the three trials, as advised in consensus statements.^10^ Manufacturers of commercial software should prioritize developing algorithms that adhere to international consensus standards and have the capability to exclude breaths/slopes that would otherwise be visually excluded by expert observers on manual review. Until this is addressed, caution should be exercised when interpreting S_cond_/S_acin_ automatically generated by software.

Researchers using the Exhalyzer D® (Eco Medics AG) for N_2_MBW should be aware that the crosstalk error between the carbon dioxide and O_2_ analyzer reported in 2021^24^ resulted in a reduction in the S_nIII_ at later TOs and thus under‐estimation of S_cond_ ^8^ in the Spiroware® software predating version 3.3.1, which incorporates a correction algorithm. Most of the Exhalyzer D® studies cited in this review will have been conducted before the correction.

### 3.7 Interpretation of results

The underlying theoretical background of S_nIII_ analysis is based on experimental and lung modeling studies in adults.^4^, ^7^, ^25^ Although the CDI and DCDI mechanisms will be identical in children, it cannot be automatically assumed either that the DCDI asymptote will occur at 1.5 TO, or that the location of the diffusion–convection front will be the same. For this reason, what S_nIII_ indices truly reflect anatomically in children with lung disease and abnormal airway structure is not fully understood.

## 4 S_NIII_ DATA IN CHILDREN WITH RESPIRATORY PATHOLOGY

In this section we summarize published S_acin_ and S_cond_ data, including their clinimetric properties in children with CF and other lung pathologies.

### 4.1 Cystic fibrosis

#### 4.1.1 Feasibility

Verger et al.^20^ applied consensus criteria and included only breaths with a volume of at least 3× fowler dead space volume for S_nIII_ analysis. The success rate was 68% (64/94) in healthy children and 63% (80/127) in children with CF (3–18 years of age). This study required at least three MBW runs meeting acceptability criteria for S_nIII_ analysis. Bigler et al.^17^ reported higher success (76%) when applying the automated algorithm in school‐age children with CF.

#### 4.1.2 Sensitivity

Children with CF have raised S_cond_ compared to healthy children ^13^, ^14^, ^20^, ^26^ with over 50% of them showing an abnormal S_cond_.^14^, ^20^, ^26^ Values of S_cond_, S_acin_, S_cond*_, and S_acin*_ reported from publications are displayed in Table 1.

**Table 1:** Summary of studies reporting MBW indices in children and adults with cystic fibrosis.

Article	Year	Gas	Age	*N*	LCI	S_cond_	S_cond*_	S_acin_	S_acin*_
Gustafsson et al.^27^	2007	N_2_	16.4	11	11.5	0.151		0.310	
Horsley et al.^13^	2008	SF_6_	12.5	18	7.3	0.068		0.192	
Singer et al.^28^	2013	N_2_	11.1	54	12.1	0.070		0.230	
Gustafsson et al.^29^	2014	N_2_	23	37	12.16	0.061		0.176	
Bigler et al.^17^	2015	N_2_	12.1^a^	35		0.060			
Nyilas et al.^26^	2016	N_2_	11.4	20	10.8	0.070		0.100	
Smith et al.^30^	2017	SF_6_	10.07	35	7.72	0.050		0.150	
Smith et al.^31^	2018	SF_6_	16.7^b^	32	10.00^b^	0.070		0.140^b^	
Nyilas et al.^14^	2018	N_2_	11.7	92	9.84	0.080	0.100	0.130	0.110
Colombo et al.^32^ ^b^	2019	N_2_	17	80	13.4	0.078		0.189	
Yammine et al^33^	2019	N_2_	9.46^a^	27	8.19^b^	0.048^b^		0.055^b^	
Skov et al.^34^ ^b^	2020	N_2_	11.6	125	10.1	0.061		0.126	
Verger et al.—preschool^20^	2020	SF_6_	4.3	86	8.6	0.058	0.067	0.110	0.110
Verger et al.—school age^20^	2020	SF_6_	13.9	41	10.6	0.072	0.100	0.19	0.18
Postek et al.^35^	2022	N_2_	12.1	20	10.16	0.060		0.120	
Pleskova et al. (intervention group)^36^	2021	N_2_	12.5	17	12.1	0.062		0.108	

*Note*: All measures are expressed in mean (or median if labelled with a small/superscript ‘b’). A small superscript ‘a’ means that age refers to all participants, including those who did not achieve S_nIII_ analysis.

Abbreviations: Gas, inert tracer gas; N, number of participants included in the study.

Studies that measured both LCI and S_cond_ in children with CF were unable to demonstrate consistently which of these two indices is more sensitive to detect early CF lung disease.^13^, ^14^, ^20^ Preschool children can have abnormal S_cond_ and S_acin_ and the indices tend to worsen with increasing age.^20^ Most commonly, abnormality is first demonstrated in S_cond,_ suggesting CF lung disease may originate in the conducting airways with convection as the primary mechanism of VI. As disease progresses, S_acin_ usually also rises,^13^ indicating patchy involvement of the peripheral lung, with elevation in S_acin_ being a later event; values are generally higher in adults than children.^13^

#### 4.1.3 Relationship with other outcomes

Only a few studies have investigated the relationship between S_nIII_ indices and structural/functional abnormality in the lung. Smith et al. assessed the association between LCI, S_cond_, and S_acin_ with hyperpolarized Helium‐3 ventilation magnetic resonance imaging (^3^He‐MRI) at end‐inspiratory tidal volume in 32 children and adults with CF.^31^ Ventilation defect percentage from ^3^He‐MRI showed a strong correlation with LCI (*r* = .89) and S_acin_ (*r* = .84) but not S_cond_ (*r* = .32), likely due to the “ceiling” effect S_cond_ observed in the presence of high ventilation inhomogeneity described earlier.

The Australian Respiratory Early Surveillance Team for CF (AREST‐CF) group studied the relationship between S_cond,_ S_acin_, phase III slope (S_III_) from single breath washout and structural changes at spirometry‐assisted volumetric chest computed‐tomography (CT), assessed using both the PRAGMA‐CF and Brody scores.^33^ Limited details were provided regarding S_nIII_ analysis QC (Supporting Information S1: E‐Table 1). While S_cond_ and LCI exhibited a significant correlation with the degree of bronchiectasis and the extent of disease, S_acin_ and S_III_ were not associated with structural damage, including air trapping extent. The CT protocol used, able to depict approximately the first six airway generations, may have missed more subtle abnormalities in the peripheral lung, which would affect DCDI and S_acin_.^33^

#### 4.1.4 Response to treatment

Gustafsson et al. performed MBW before and after nebulization of a short‐acting beta2‐agonist (salbutamol) in a small sample size of 11 children with CF.^27^ The LCI and S_cond_ did not change while S_acin_ improved (*p* < .01); however, all indices remained abnormal post‐bronchodilation.

#### 4.1.5 Sensitivity of S_cond*_ and S_acin*_

Two studies have reported on the use of S_cond*_ and S_acin*_ in children with CF.^14^, ^20^ In both studies, researchers used in‐house software to conduct visual breath‐by‐breath QC and determine values based on consensus criteria.^10^ Nyilas et al. found that, using N_2_MBW, S_cond_ was less sensitive than LCI to detect CF lung disease in a cohort of 92 Swiss patients with CF (mean ± SD 11.7 ± 3.9 years), with 87% (80/92) of them having an abnormal LCI but only 60% (55/92) showing an abnormal S_cond_. Since most patients had mild to moderate CF lung disease (mean ± SD LCI = 9.84 ± 1.85), it is not surprising that the alternative indices S_cond*_ and S_acin*_ demonstrated even lower sensitivity, being abnormal in 19% (17/92) and 12% (11/92) of the cohort, respectively.^14^

The London CF Collaboration (LCFC) assessed S_cond*_ and S_acin*_ in a large cohort of 127 children ranging from 3 to 18 years, who performed SF_6_ MBW.^20^ Compared to Nyilas et al.,^14^ they found a higher frequency of S_cond_ abnormality (69% vs. 60%). The proportion of patients with an abnormal S_cond*_ was greater in the LCFC than in the Swiss study (52% vs. 19%), most likely because the former included sicker patients (mean ± SD LCI = 8.62 ± 1.93 at preschool and 10.62 ± 3.07 at school age). In patients with moderate to severe VI (LCI ≥ 9), there was a greater correlation of LCI with S_cond*_ rather than with S_cond_.

In summary, current evidence suggests that S_nIII_ could integrate LCI in the early tracking of CF lung disease, although feasibility is limited at school age; moreover, more data from longitudinal studies and a better definition of minimal clinically meaningful changes in S_nIII_ outcomes are needed. It is also evident that S_cond_ has very limited value in CF patients with moderate to high ventilation inhomogeneity (i.e., LCI ≥ 9).

### 4.2 Primary ciliary dyskinesia

A few studies have investigated S_nIII_ indices in children with primary ciliary dyskinesia (PCD).^15^, ^37^, ^38^ Abnormalities in S_cond_ and S_acin_ were highly prevalent in this group even in children with normal spirometry.^15^, ^37^ Green et al.^37^ reported S_cond_ reached a plateau at LCI values around +10 *z*‐scores. It is possible that this finding is related to the known limitation of S_cond_ in severe VI. Consistent with this, Nyilas et al.^15^ showed improved agreement between S_cond*_ and LCI and FEV_1_ over standard S_cond_, in their cohort of 49 children with PCD and moderate to severe VI (mean ± SD LCI 11 ± 3.6, range 7.0–23).

Kobbernagel et al.^38^ report the only published longitudinal study of S_cond_ and S_acin_ in pediatric PCD. MBW data were collected at three different data points in 42 children and young adults over 1 year (median age 15.4 years, age 6–29). S_cond_/S_acin_ were derived using commercial software, however with breath‐by‐breath quality control before calculations (Supporting Information S1: E‐Table 1). Overall, both mean S_cond_ and S_acin_ remained stable over the course of the study while the average LCI had a mild but significant increase by 0.5 points (95% CI: 0.12, 0.91; *p* = .01). The study was not powered to assess longitudinal changes of LCI or S_nIII_ indices; therefore, findings should be interpreted with caution. However, there was a quite high variability in either LCI, S_acin_, and S_cond_, suggesting that MBW is not an ideal measure to track closely the evolution of chronic lung disease over the time in PCD, because it would be difficult to define a minimal clinically meaningful change in these outcomes.

### 4.3 Asthma

Conductive and acinar VI is present in adults with asthma.^39^

Conductive VI is also a feature of childhood asthma^27^, ^40^, ^41^ and it has been occasionally reported in preschool children with multi‐trigger wheezing.^42^, ^43^, ^44^

Gustafsson et al. using custom‐made N_2_MBW, found similarly elevated S_cond_ values in 15 school‐age children with moderate to severe asthma (mean ± SD FEV_1_ 77 ± 14% predicted) and 11 children with CF.^27^ In the asthma group, S_cond_ improved but remained abnormal with bronchodilator response (BDR), suggesting chronic airways impairment and remodeling. Lack of response in S_cond_ was also reported in children with severe therapy‐resistant asthma after bronchoscopy and intramuscular injection of triamcinolone.^45^ In a cohort of 31 children with milder asthma (mean ± SD FEV_1_ −1.09 ± 1.28 *z*‐scores), Macleod et al.^41^ detected only a trend toward higher S_cond_ values compared to healthy controls (mean ± SD S_cond_ 0.026 ± 0.02 vs. 0.017 ± 0.02; *p* = .06), while LCI was significantly higher in the asthma group (6.67 ± 0.91 vs. 6.24 ± 0.47).

Steinbacher et al.^46^ compared lung function outcomes pre‐ and post‐indirect airway challenge (cold dry air) in 43 children (range 6.5–18.6 years) with a previous history of asthma. LCI and S_cond_ significantly increased post‐challenge in children with airway hyper‐responsiveness (AHR), as assessed through spirometry. In 47 children with active allergic asthma, instead, Keen et al.^40^ showed that AHR by spirometry was associated with higher baseline S_cond_ and bronchial nitric oxide (NO) (a marker of eosinophilic airway inflammation). They also found 38% of the asthmatic children had abnormal S_acin;_ however, it is not clear whether breath‐by‐breath QC before S_nIII_ analysis was performed. Abnormal S_acin_ was also reported in a later study of 42 children with asthma (6–17 years). Higher prevalence for abnormality was found in the “asthma exacerbation” group (76% [15/20]) compared to stable children (27% [6/22]).^47^ Prospective longitudinal studies are needed to assess whether S_acin_ or S_cond_ can be a useful marker to assess response to asthma treatment and/or predict future exacerbations.

Specific measures of VI have been reported in studies of preschool children with recurrent wheezing. S_cond_ was the lung function parameter more frequently abnormal (43% of the cohort) in 34 children with severe multi‐trigger wheezing aged 4–6 years, who also underwent FeNO and specific airways resistance^44^ and was not fully reversible with bronchodilator therapy suggesting the possibility of structural changes with airway remodeling.

### 4.4 Chronic lung disease of prematurity

In 77 preterm children (mean gestational age [GA] 28 weeks, range 23–34 weeks) who underwent N_2_MBW at school age, S_cond_ was significantly higher than in healthy controls born at term (0.031 ± 0.012 vs. 0.017 ± 0.011; *p* < .001) and, among those born before 28 weeks of gestation, there was a negative association between GA and S_cond_ values.^12^ There were no statistically significant differences between preterm and term‐born children in mean LCI and S_acin_ values.^12^ Using the same approach to S_nIII_ analysis with breath‐by‐breath QC, Arigliani et al.^11^ reported outcomes in preterm children via commercial software. S_cond_ abnormalities were found in 29% (13/44) of preterm children born <28 weeks GA, while only 14% (6/44) of them had abnormal FEV_1_ (*p* = .06) and 16% (7/44) had abnormal S_acin_. A history of BPD was not associated with higher S_cond_, as also reported by Yammine et al.^12^ Sørensen et al.^48^ reported S_cond_ values similar to these two studies in a cohort of 70 school‐age children born <28 weeks GA but did not detect a significant difference in S_cond_ between controls and preterm.

Overall, these findings suggest the presence of conductive VI associated with prematurity, particularly in those born <28 weeks GA. This could be related to an impaired lung parenchymal elastic network development, affecting the tethering of the airways and their mechanics with a patchy distribution.^49^ Encouragingly, in recent cohorts of extreme preterm children, S_acin_ is frequently normal, suggesting postnatal catch‐up alveolarization with a limited functional impact on the more peripheral lung.^11^

### 4.5 Other chronic respiratory disorders

A single‐center cross‐sectional study reported regional VI indices in children and young adults with allogeneic hematopoietic stem cell transplantation (HSCT).^50^ Although measurements were undertaken with Exhalyzer D®, S_nIII_ analysis was performed using an in‐house software (Supporting Information S1: E‐Table 1 for more details). Sixty‐four patients (mean age 14.9 years, range 7.9–24), including one with known bronchiolitis obliterans syndrome, and 64 healthy controls, underwent MBW 3–10 years post HSCT. Over half of the subjects (52%) had abnormal S_cond_ while only 9% had abnormal FEV_1_. In particular, 9 out of 11 subjects with extra‐pulmonary graft‐versus‐host disease (GvHD) had S_cond_ > 97.5th percentile of the control population, with three of them also having abnormal FEV_1_. S_acin_ was abnormal in 25% of patients. High LCI and S_cond_ values were associated with more frequent respiratory symptoms. Further longitudinal studies are needed to assess whether S_cond_ abnormalities in post‐HSCT children could be an early marker of lung GvHD.

A cross‐sectional study on an unselected cohort of 35 children and adolescents with sickle cell anemia (mean ± SD age 16.4 ± 3.5 years) found normal S_cond_ values but significantly higher LCI and S_acin_ compared to healthy controls, suggesting peripheral lung impairment.^51^ Further longitudinal studies are necessary to determine whether LCI and S_acin_ can be early markers of chronic sickle‐cell‐related lung disease, anticipating the onset of restrictive lung function defects, more commonly seen in adults with this condition.

## 5 LIMITATIONS

This narrative review highlights the poor standardization when S_cond_/S_acin_ is applied as outcomes measures in studies. While some research groups reported S_cond_/S_acin_ values after rigorous breath‐by‐breath QC measures, others appear to rely on automated software (see Supporting Information S1: E‐Table 1 for details), hindering interpretation of results.

The lack of standardization along with the high variability and numerous gaps for research indicate that, currently, S_cond_ and S_acin_ do not possess the same robust clinimetric properties observed with LCI. Moreover, since S_nIII_ outcomes and LCI correlate to some extent ^13^, ^20^ they may provide in part overlapping information. However, only S_cond_ and S_acin_, but not LCI, give insight into the source of ventilation inhomogeneity along the respiratory tract, which can represent very useful data from a clinical perspective. Since this was a narrative review, we did not analyze systemically biases of the study included but we have critically analyzed findings, including study limitations. Some of the studies discussed in the review had small sample sizes and were not adequately powered, limiting the value of their results (Supporting Information S1: E‐Table 1).

## 6 CONCLUSION

S_nIII_ indices have the potential to reveal regional VI, which can help to localize the site of impairment along the respiratory tree in people with gas mixing deficits. If used in early childhood, S_nIII_ indices may provide an indication of the origin or pathological patterns of respiratory disease. In children with CF, S_cond_ alteration can even precede LCI abnormalities and can help to track early CF lung disease over the time. In other conditions, like PCD, asthma, or chronic lung disease of prematurity where S_nIII_ indices have been reported to be often abnormal in cross‐sectional studies, it remains to be established if they have value in tracking progress longitudinally or in assessing response to treatment.

Further work is required to improve standardization and QC, and to establish reference values and minimal clinically important differences. Unless these issues are addressed, it is likely the use of S_nIII_ indices in pediatrics will remain largely within the research setting.

## AUTHOR CONTRIBUTIONS

**Mollie Riley**: Conceptualization; data curation; formal analysis; writing—original draft; writing—review & editing. **Michele Arigliani**: Conceptualization; formal analysis; writing—original draft; writing—review and editing. **Gwyneth Davies**: Conceptualization; formal analysis; writing—review and editing. **Paul Aurora**: Conceptualization; formal analysis; writing—review and editing.

## CONFLICTS OF INTEREST STATEMENT

Mollie Riley reports speaker honoraria from Vertex Pharmaceuticals outside of this submitted work. Gwyneth Davies reports speaker honoraria from Chiesi Ltd and Vertex Pharmaceuticals, and advisory board and clinical trial leadership roles with Vertex Pharmaceuticals, outside of this submitted work. The remaining authors declare no conflict of interest.

## Supporting information

Supporting information.

## Data Availability

Data sharing is not applicable to this article as no datasets were generated or analyzed during the current study.
