# Supplementary material for: Looking beyond LCI: Multiple breath washout phase III slope derived indices and their application in chronic respiratory disease in children
Source: Pediatr Pulmonol. 2024 Jul 19;59(12):3085–94. doi: 10.1002/ppul.27177 (PMC11601015; doi:10.1002/ppul.27177)
Supplement: Supplementary file 1 — Supporting information. [file PPUL-59-3085-s001.docx]

Looking beyond LCI: Multiple Breath Washout phase III slope derived indices and their application in chronic respiratory disease in children.

Authors: Riley, Mollie BSc^1,2^., Arigliani, Michele MD^1,3^., Davies., Gwyneth MBChB PhD^2,4^., Aurora., Paul MBBS, PhD^1,2^.

Affiliations:

1. Infection, Immunity and Inflammation Research and Teaching Dept, UCL Great Ormond Street Institute of Child Health (UCL GOS ICH), London, UK.
2. Heart and Lung Directorate, Great Ormond Street Hospital for Children NHS Foundation Trust, London, UK.
3. Department of Respiratory Paediatrics, Royal Brompton Hospital, London, UK.
4. Population, Policy and Practice Research and Teaching Dept, UCL GOS ICH, London, UK.

**E-Table 1:** Summary of S_cond_/S_acin_ findings and the quality control approaches used in paediatrics

| Article | Sample | Key S_nIII_ findings | MBW methodology | Interval for defining S_nIII_ | Breath-by-Breath QC | Derivation of S_cond_/S_acin_ | Interpretation |
| --- | --- | --- | --- | --- | --- | --- | --- |
| Gustafsson et al 2007(1) | 11 CF  15 Asthma  18 HC | S_acin_ higher in CF than asthma (p<0.01) but S_cond_ similar. | Custom-made N_2_ washout. | 65-95% of expired volume. | vT/FRC ratios checked to ensure large enough breath size. | Used Verbanck 1997 method (2). | Limited QC information. |
| Horsley et al 2008 (3) | Children: 18 CF, 29 HC  Adults: 22 CF, 17 HC | S_cond_ abnormal in CF and reaches a maximum (0.150). | SF_6_ washout, Innocor and Testpoint (Capital Equipment, Massachusetts, USA). | Manual identification. | Visual QC performed. | Used Verbanck 1997 method (2) (but with S_nIII_ x vT) however, each trial analysed separately and average S_cond_/S_acin_ reported. | Strict manual QC. |
| Singer et al 2013 (4) | 51 CF  44 HC | SBW S_III_ correlated with S_acin_ (r=-0.58; p<0.001), but not with S_cond_ (r =-0.03). | N_2_ washout, Exhalyzer D® and Spiroware 3.1 and WBreath®, 3.28 (ndd Medical Technologies, Zurich, Switzerland). | Not stated. | Not stated. | Limited information. | S_cond_/S_acin_ appears to be determined using WBreath® software. |
| Bigler et al 2015 (5) | 35 CF  20 HC | Scond_auto_ same as Scond_manual_ with comparable fitting (CF R^2^ =0.53 and HC =0.13 vs. CF R^2^ =0.54 and HC =0.13). | Exhalyzer D® and Spiroware 3.1.6 and custom-made software (based on MATLAB). | Scond_auto_:65-95%.  Scond_manual_: manual identification. | Scond_manual_: visual QC.  Scond_auto_: excluded breaths with a vT deviating >25% from median vT. | Scond_manual_: calculations based on consensus (6). Scond_auto_: Each trial analysed separately, average S_cond_/S_acin_ reported. Trials with <2/3 of breaths left after QC or with an inadequate first breath were discarded. | Automated algorithm and strict manual QC used. |
| Nyilas et al 2016 (7) | 20 CF  20 PCD  20 preterm  20 HC | Using clustering methods, 3 phenotypes found:  I: normal outcomes, II: increased S_cond_, normal S_acin_ and III: abnormal S_cond_ and S_acin._ | Exhalyzer D®, (software unknown). | 65-95%. | Visual QC performed. | Limited information. | S_cond_/S_acin_ appears to be determined semi-automatically. |
| Smith et al 2017 (8) | 35 CF  28 HC | Inconsistent changes in S_cond_/S_acin_ from sitting to supine in CF/HC. | SF_6_ washout, Innocor and custom-built software. | Manual identification. | Visual QC performed. | Calculations based on consensus (6). | Strict manual QC. |
| Smith et al 2018 (9) | 32 CF | VDP correlated with S_acin_ (r=0.84; p<0.001), but not S_cond_ (r =0.32). | SF_6_ washout, Innocor and custom-built software. | Not stated. | Not stated. | Not stated. | No QC information. |
| Nyilas et al 2018 (10) | 92 CF  43 HC | In CF, S_cond_ abnormal in 60%, S_acin_ in 11%. S_cond_*/S_acin*_ less sensitive. ICC for LCI, S_cond_ and S_cond_* was 0.9, 0.6 and 0.1. ICC higher in patients with CF than HC. | N_2_ washout, Exhalyzer D® and in-house software (based on MATLAB). | 65-95%. | Visual QC performed. | Calculations based on consensus (6). Only trials in which at least six S_nIII_ data points used. | S_cond_/S_acin_ appears to be determined semi-automatically. |
| Colombo et al 2019 (11) | 80 CF | β-cell glucose sensitivity was inversely associated with S_cond_ (r=−0.36; p<0.01). | N_2_ washout, Exhalyzer D® and Spiroware 3.2. | Not stated. | Not stated. | Not stated. | S_cond_/S_acin_ appears to be reported directly from automated values on Spiroware. |
| Yammine et al 2019 (12) | 27 CF | S_cond_ associated with bronchiectasis extent on CT (r=0.60, p<0.01). | N_2_ washout, Exhalyzer D® and Spiroware 3.1.6. | Not stated. | Not stated. | Limited information. | S_cond_/S_acin_ potentially reported directly from automated values on Spiroware. |
| Skov et al 2020 (13) | 125 CF  177 HC | In CF, specific ventilation of the slower compartment inversely correlated with S_acin_ (r^2^=−0.36; p<0.01) and S_cond_ (r^2^=−0.51; p<0.0001). | N_2_ washout, Exhalyzer D® and Spiroware 3.1 and TestPoint. | Not stated. | Not stated. | Brief mention of QC “S_nIII_ analysis was performed as previously described by Aurora et al 2005” (14). | Limited QC information. |
| Verger et al 2020 (15) | 127 CF  94 HC | S_cond_, S_cond*_, S_acin_, S_acin*_ worsening trend with age in CF. S_cond_ reaches a maximum at ~LCI 9. | SF_6_ washout, respiratory mass spectrometer and TestPoint. | 55-95%. | Visual QC performed. Breaths with expiratory vT <3x median Fowler dead space volume were excluded. | Calculations based on consensus (6) including - trials with <2/3 of breaths left after QC were discarded. | Strict manual QC. |
| Pleskova et al 2021 (16) | 17 CF | After reflex zone stimulation technique (RST), LCI and S_cond_ decreased (p<0.01), S_acin_ did not. | N_2_ washout, Exhalyzer D® (software unknown). | 65-95%. | Visual QC performed. | Includes consensus criteria (6) - trials with <2/3 of breaths left after QC were discarded. | S_cond_/S_acin_ appears to be determined semi-automatically. |
| Postek et al 2022 (17) | 20 CF | S_cond_ but not S_acin_ correlated with impulse oscillometry (R5Hz=r =0.56 and R20Hz =0.55; no p value reported), and FEV_1_/FVCz-score (r=−0.62; no p value reported). | N_2_ washout, Exhalyzer D® and Spiroware 3.2. | Not stated. | Not stated however, S_cond_/S_acin_ reported from subjects with vT between 10-15ml/kg. | Not stated. | S_cond_/S_acin_ appears to be reported directly from automated values on Spiroware. |
| Yammine et al 2014 (18) | 15 CF  20 HC | Fixed 1-Litre breathing increased S_cond_ (p<0.01). S_acin_ remained unchanged. | Exhalyzer D® and Spiroware 3.1.6. | 65-95%. | Visual QC performed. | Reported from Spiroware but states ‘outcomes calculated as currently recommended’. | S_cond_/S_acin_ appears to be determined semi-automatically. |
| Arigliani et al 2020 (19) | 47 preterm  60 controls | Abnormal S_cond_ found in 29% of extremely preterm children at school age. | Exhalyzer D® and Spiroware 3.1.6. | 55-95%. | Visual QC performed including breaths with a S_III_ volume <50% or >75% of the total expired volume, were excluded. | Reported from Spiroware but includes consensus criteria (6) - trials with <2/3 of breaths left after QC were discarded. | S_cond_/S_acin_ appears to be determined semi-automatically. |
| Yammine et al 2016 (20) | 77 preterm  46 HC | S_cond_ increased in preterm compared to HC (p<0.001). No difference in S_acin_. Abnormal S_cond_ found in 54% of preterm children. | Exhalyzer D® and Spiroware 3.1.6. | 65-95. | Visual QC performed. | Reported from Spiroware but includes consensus criteria (6) - trials with <2/3 of breaths left after QC were discarded. | S_cond_/S_acin_ appears to be determined semi-automatically. |
| Green et al 2012 (21) | 23 PCD | S_cond_ and S_acin_ abnormal in 96% and 78% in PCD. LCI correlated with S_acin_ (R^2^=0.45; p<0.001), not S_cond_. | SF_6_ washout, respiratory mass spectrometer and TestPoint. | Not stated. | Not stated. | Calculations based on consensus (6). | Limited QC information. |
| Kobbernagel et al 2019 (22) | 42 PCD | LCI increased (p<0.05), but S_cond_/S_acin_ did not change over 1 year. LCI, S_acin_ (not S_cond_) showed strong ICC ranging from 0.76 to 0.86. | N_2_ washout, Exhalyzer D® and Spiroware 3.1.6. | Not stated. | Visual QC performed. | Reported from Spiroware. | S_cond_/S_acin_ appears to be determined semi-automatically. |
| Nyilas et al 2017 (23) | 49 PCD  37 HC | S_cond_ abnormal in a similar proportion to LCI (78% and 79%, respectively). Variability of S_acin_, S_cond_ was between 24% - 76% and higher in HC than PCD. | N_2_ washout, Exhalyzer D® and Spiroware 3.1.6. | Not stated. | Not stated. | Reported from Spiroware. | Limited QC information. |
| Keen et al 2011 (24) | 47 asthma  36 HC | In asthma, S_cond_ abnormal in 31 (66%) and S_acin_ in 18 (38%). Subjects with AHR had higher S_cond_ than those without (p=0.001). | SF_6_ washout, respiratory mass spectrometer and TestPoint. | Not stated. | Not stated however, S_cond_/S_acin_ reported from subjects with vT between 10-15ml/kg. | Limited information. | Limited QC information but did state several HC did not meet QC criteria. |
| Macleod et al 2008 (25) | 31 asthma  28 HC | No difference in S_acin_ between asthma and HC but a trend towards higher S_cond_ in asthmatics (p=0.06). No change in S_cond_ or S_acin_ post-BD. | SF_6_ washout, Innocor and TestPoint. | Not stated. | Not stated. | Used Verbanck 1997 method (2). | Limited QC information. |
| Racette et al 2018 (26) | 25 CF  33 HC | Higher S_cond_ in subjects with a history of severe asthma compared with HC (p=0.02). | SF_6_ washout, respiratory mass spectrometer and TestPoint. | Not stated. | Visual QC performed. | Calculations based on consensus (6) including - trials with <2/3 of breaths left after QC were discarded. | Strict manual QC. |
| Vilmann et al 2017 (27) | 35 asthma  30 HC | S_cond_ higher in asthma compared to HC (p<0.01). No difference in S_acin_.. | N_2_ washout, Exhalyzer D® and Spiroware 3.1.6. | Not stated. | Not stated. | Reported from Spiroware. | S_cond_/S_acin_ appears to be reported directly from automated values on Spiroware. |
| Sonnappa et al 2010 (28) | 28 episodic wheezers, 34 multiple-trigger wheezers  72 HC | S_cond_ greater sensitivity than LCI (68% vs 39%). | SF_6_ washout, respiratory mass spectrometer. | Not stated. | Not stated. | States “S_cond_/ S_acin_ were estimated by calculating S_III_, as previously described in Aurora 2005 (14). | No information on QC. |
| Steinbacher et al 2017 (29) | 43 asthma | Subjects with AHR showed increase in S_cond_ (p<0.01) after cold dry air challenge (CACh) and decreases after salbutamol (p<0.01). Normoresponsive subjects showed no change after CACh, but a decrease after salbutamol (p<0.01). | N_2_ washout, Exhalyzer D® and Spiroware 3.1.6. | 65-95%. | Visual QC performed. | Reported from Spiroware but includes consensus criteria (6) - trials with <2/3 of breaths left after QC were discarded. | S_cond_/S_acin_ appears to be determined semi-automatically. |
| Wawszczak et al 2022 (30) | 22 stable asthma, 20 asthma with exacerbation | Stable asthma: Abnormal S_cond_/S_acin_ in 23% and 27%. Asthma exacerbation abnormal S_cond_/S_acin_ in 100% and 76%. | N_2_ washout, Exhalyzer D® (software unknown) | Not stated. | Not stated. | Reported from Spiroware. | No QC information. |
| Irving et al 2020 (31) | 21 difficult asthma (DA), 43 severe therapy‐resistant asthma (STRA)  21 HC | S_cond*_ was different between DA and STRA (p<0.005) and STRA and HC (p<0.0006); but overlap present. S_acin*_ was normal even in STRA. S_cond*_ did not improve in STRA after bronchoscopy and triamcinolone injection. | SF_6_ washout, Innocor and Testpoint. | Not stated. | Visual QC performed. | Calculated S_cond*_ and S_acin*_ using in-house software, results were excluded if more than 3 breaths over the analysis section (to turnover 3) were unusable. | Strict manual QC, based on consensus. |
| Sørensen et al 2018 (32) | 42 BPD  28 non-BPD  38 HC | S_acin_ higher in both BPD and non-BPD compared to HC (p<0.01). No difference seen in S_cond_. | N_2_ washout, Exhalyzer D® and Spiroware 3.1.6. | Not stated. | Not stated. | Reported from Spiroware. | No QC information. |
| Uhlving et al 2015 (33) | 64 HSCT-subjects  64 HC | In HSCT, S_acin_ was abnormal in 25% and S_cond_ in 52%. S_cond_ was abnormal in 82% (9/11) of chronic GvHD subjects. | N_2_ washout, Exhalyzer D® and Spiroware 3.1.5 and TestPoint. | Not stated. | Not stated. | Calculations based on consensus (6). | Limited QC information. |
| Arigliani et al 2022 (34) | 35 SCA  31 HC | S_acin_ higher in SCA compared to HC (p=0.04). No difference in S_cond_. | N_2_ washout, Exhalyzer D® and data re-analysed using Spiroware 3.3.1. | 55-95%. | Visual QC performed including breaths with a S_III_ volume <50% or >75% of the total expired volume, were excluded. | Reported from Spiroware but includes consensus criteria (6) - trials with <2/3 of breaths left after QC were discarded. | S_cond_/S_acin_ appears to be determined semi-automatically. |
| Sisman et al 2016 (35) | 22 Hypersensitivity Pneumonitis | S_cond_ abnormal in 53%, and S_acin_ in 32%, FEV_1_ was only abnormal in 9%. | N_2_ washout, Exhalyzer D® and Spiroware 3.1.6. | Not stated. | Not stated. | Reported from Spiroware but states measured as per current consensus (6). | S_cond_/S_acin_ appears to be determined semi-automatically |
| Yammine et al 2016 (36) | 28 inflammatory bowel disease (IBD)  31 HC | Subjects with IBD show similar LCI, S_cond_ and S_acin_ to HC. | N_2_ washout, Exhalyzer D® and Spiroware 3.1.6. | Not stated. | Visual QC performed. | Reported from Spiroware but states measured as per current consensus (6). | S_cond_/S_acin_ appears to be determined semi-automatically. |

***Legend:*** *Abbreviations: AHR: Airway Hyperresponsiveness, CACh: Cold Dry Air Challenge, CF: Cystic Fibrosis, DA: Difficult Asthma, FEV_1_: Forced Expiratory Volume in one second, FVC: Forced Vital Capacity, HC: Healthy Controls, IBD: Inflammatory Bowel Disease, ICC: Intraclass Correlation Coefficient, LCI: Lung Clearance Index, MBW: Multiple Breath Washout, N_2_: Nitrogen, PCD: Primary Ciliary Dyskinesia, QC: Quality Control, R5Hz: resistance at low frequencies, R20Hz: resistance at high frequencies, SBW: Single Breath Washout, Scond_auto:_ S_cond_ calculated using the Exhalyzer D automated algorithm, Scond_all:_ S_cond_ calculated using all breaths of the 3 trials, Scond_manual:_ S_cond_ calculated using the traditional/manual method, SD: Standard Deviation, SF_6_: Sulphur Hexafluoride, S_III_: Phase III Slope, S_nIII_: Normalised Phase III Slope, STRA: Severe Therapy‐Resistant Asthma, vT: Tidal Volume.*

**References:**

1. Gustafsson PM. Peripheral airway involvement in CF and asthma compared by inert gas washout. Pediatr Pulmonol. 2007 Feb;42(2):168–76.

2. Verbanck S, Schuermans D, Van Muylem A, Paiva M, Noppen M, Vincken W. Ventilation distribution during histamine provocation. J Appl Physiol 1985. 1997 Dec;83(6):1907–16.

3. Horsley AR, Macleod KA, Robson AG, Lenney J, Bell NJ, Cunningham S, et al. Effects of cystic fibrosis lung disease on gas mixing indices derived from alveolar slope analysis. Respir Physiol Neurobiol. 2008 Aug 31;162(3):197–203.

4. Singer F, Stern G, Thamrin C, Abbas C, Casaulta C, Frey U, et al. A new double-tracer gas single-breath washout to assess early cystic fibrosis lung disease. Eur Respir J. 2013 Feb;41(2):339–45.

5. Bigler A, Yammine S, Singer F, Riedel T, Latzin P. Feasibility of automated slope III and Scond analysis in children: Automated Scond Calculation in Children. Pediatr Pulmonol. 2015 Aug;50(8):805–13.

6. Robinson PD, Latzin P, Verbanck S, Hall GL, Horsley A, Gappa M, et al. Consensus statement for inert gas washout measurement using multiple- and single- breath tests. Eur Respir J. 2013 Mar;41(3):507–22.

7. Nyilas S, Singer F, Kumar N, Yammine S, Meier-Girard D, Koerner-Rettberg C, et al. Physiological phenotyping of pediatric chronic obstructive airway diseases. J Appl Physiol. 2016 Jul 1;121(1):324–32.

8. Smith LJ, Macleod KA, Collier GJ, Horn FC, Sheridan H, Aldag I, et al. Supine posture changes lung volumes and increases ventilation heterogeneity in cystic fibrosis. Singer F, editor. PLOS ONE. 2017 Nov 27;12(11):e0188275.

9. Smith LJ, Collier GJ, Marshall H, Hughes PJC, Biancardi AM, Wildman M, et al. Patterns of regional lung physiology in cystic fibrosis using ventilation magnetic resonance imaging and multiple-breath washout. Eur Respir J. 2018 Nov;52(5):1800821.

10. Nyilas S, Bigler A, Yammine S, Kieninger E, Rochat I, Ramsey K, et al. Alternate gas washout indices: Assessment of ventilation inhomogeneity in mild to moderate pediatric cystic fibrosis lung disease. Pediatr Pulmonol. 2018 Nov;53(11):1485–91.

11. Colombo C, Alicandro G, Gambazza S, Mileto P, Mari A, Grespan E, et al. Ventilation inhomogeneity is associated with OGTT-derived insulin secretory defects in cystic fibrosis. Pediatr Pulmonol. 2019;54(2):141–9.

12. Yammine S, Ramsey KA, Skoric B, King L, Latzin P, Rosenow T, et al. Single‐breath washout and association with structural lung disease in children with cystic fibrosis. Pediatr Pulmonol. 2019 May;54(5):587–94.

13. Skov L, Green K, Stanojevic S, Jensen R, Buchvald F, Ratjen F, et al. Lung compartment analysis assessed from N _2_ multiple‐breath washout in children with cystic fibrosis. Pediatr Pulmonol. 2020 Jul;55(7):1671–80.

14. Aurora P, Kozlowska W, Stocks J. Gas mixing efficiency from birth to adulthood measured by multiple-breath washout. Respir Physiol Neurobiol. 2005 Aug 25;148(1–2):125–39.

15. Verger N, Arigliani M, Raywood E, Duncan J, Negreskul Y, Bush A, et al. Limitations of regional ventilation inhomogeneity indices in children with cystic fibrosis. Pediatr Pulmonol. 2020 Sep;55(9):2315–22.

16. Pleskova J, Koucky V, Medunova K, Vlckova B, Smolikova L, Pohunek P. Reflex zone stimulation reduces ventilation inhomogeneity in cystic fibrosis: A randomised controlled cross‐over study. Pediatr Pulmonol. 2021 Jun;56(6):1558–65.

17. Postek M, Walicka-Serzysko K, Milczewska J, Sands D. What Is Most Suitable for Children With Cystic Fibrosis—The Relationship Between Spirometry, Oscillometry, and Multiple Breath Nitrogen Washout. Front Pediatr. 2022 Jan 14;9:692949.

18. Yammine S, Singer F, Gustafsson P, Latzin P. Impact of different breathing protocols on multiple-breath washout outcomes in children. J Cyst Fibros. 2014 Mar;13(2):190–7.

19. Arigliani M, Valentini E, Stocco C, De Pieri C, Castriotta L, Barbato V, et al. Regional ventilation inhomogeneity in survivors of extremely preterm birth. Pediatr Pulmonol. 2020 Jun;55(6):1366–74.

20. Yammine S, Schmidt A, Sutter O, Fouzas S, Singer F, Frey U, et al. Functional evidence for continued alveolarisation in former preterms at school age? Eur Respir J. 2016 Jan;47(1):147–55.

21. Green K, Buchvald FF, Marthin JK, Hanel B, Gustafsson PM, Nielsen KG. Ventilation inhomogeneity in children with primary ciliary dyskinesia. Thorax. 2012;67(1):49–53.

22. Kobbernagel HE, Green K, Ring AM, Buchvald FF, Rosthøj S, Gustafsson PM, et al. One-year evolution and variability in multiple-breath washout indices in children and young adults with primary ciliary dyskinesia. Eur Clin Respir J. 2019 Jan 1;6(1):1591841.

23. Nyilas S, Schlegtendal A, Singer F, Goutaki M, Kuehni CE, Casaulta C, et al. Alternative inert gas washout outcomes in patients with primary ciliary dyskinesia. Eur Respir J. 2017 Jan;49(1):1600466.

24. Keen C, Olin AC, Wennergren G, Gustafsson P. Small airway function, exhaled NO and airway hyper-responsiveness in paediatric asthma. Respir Med. 2011 Oct;105(10):1476–84.

25. Macleod KA, Horsley AR, Bell NJ, Greening AP, Innes JA, Cunningham S. Ventilation heterogeneity in children with well controlled asthma with normal spirometry indicates residual airways disease. Thorax. 2008 Oct 3;64(1):33–7.

26. Racette C, Lu Z, Kowalik K, Cheng O, Bendiak G, Amin R, et al. Lung clearance index is elevated in young children with symptom-controlled asthma. Health Sci Rep. 2018 Aug;1(8):e58.

27. Vilmann L, Buchvald F, Green K, Nielsen KG. Fractional exhaled nitric oxide and multiple breath nitrogen washout in preschool healthy and asthmatic children. Respir Med. 2017 Dec;133:42–7.

28. Sonnappa S, Bastardo CM, Wade A, Saglani S, McKenzie SA, Bush A, et al. Symptom-pattern phenotype and pulmonary function in preschool wheezers. J Allergy Clin Immunol. 2010 Sep;126(3):519-526.e7.

29. Steinbacher M, Pfleger A, Schwantzer G, Jauk S, Weinhandl E, Eber E. Small airway function before and after cold dry air challenge in pediatric asthma patients during remission. Pediatr Pulmonol. 2017 Jul;52(7):873–9.

30. Wawszczak M, Kulus M, Peradzyńska J. Peripheral airways involvement in children with asthma exacerbation. Clin Respir J. 2022 Feb;16(2):97–104.

31. Irving S, Fleming L, Ahmad F, Biggart E, Bingham Y, Cook J, et al. Lung clearance index and steroid response in pediatric severe asthma. Pediatr Pulmonol. 2020 Apr;55(4):890–8.

32. Sørensen JK, Buchvald F, Berg AK, Robinson PD, Nielsen KG. Ventilation inhomogeneity and NO and CO diffusing capacity in ex-premature school children. Respir Med. 2018 Jul;140:94–100.

33. Uhlving HH, Mathiesen S, Buchvald F, Green K, Heilmann C, Gustafsson P, et al. Small airways dysfunction in long-term survivors of pediatric stem cell transplantation: Multiple Breath Washout in Stem Cell Recipients. Pediatr Pulmonol. 2015 Jul;50(7):704–12.

34. Arigliani M, Kirkham FJ, Sahota S, Riley M, Liguoro I, Castriotta L, et al. Lung Clearance Index May Detect Early Peripheral Lung Disease in Sickle Cell Anemia. Ann Am Thorac Soc. 2022;19(9):1507–15.

35. Sisman Y, Buchvald F, Blyme AK, Mortensen J, Nielsen KG. Pulmonary function and fitness years after treatment for hypersensitivity pneumonitis during childhood. Pediatr Pulmonol. 2016 Aug;51(8):830–7.

36. Yammine S, Nyilas S, Casaulta C, Schibli S, Latzin P, Sokollik C. Function and Ventilation of Large and Small Airways in Children and Adolescents with Inflammatory Bowel Disease: Inflamm Bowel Dis. 2016 Aug;22(8):1915–22.
